# Successful Aging at Work: Psychometric Properties of the Spanish Version of Selection, Optimization and Compensation Questionnaire

**DOI:** 10.3389/fpsyg.2018.00410

**Published:** 2018-04-04

**Authors:** Adrián Segura-Camacho, Francisco Rodríguez-Cifuentes, Luis C. Sáenz De la Torre, Gabriela Topa

**Affiliations:** ^1^Department of Developmental and Educational Psychology, University of Huelva, Huelva, Spain; ^2^Christian Charities Association, Huelva, Spain; ^3^Department of Social and Organizational Psychology, Universidad Nacional de Educación a Distancia, Madrid, Spain

**Keywords:** successful aging, selection, optimization and compensation, adaptive performance, organizational citizenship behaviors

In developed countries, the aging population poses a challenge to the management of human resources in organizations (Yenilmez, 2015). On the one hand, there are a growing number of workers who are aging at the same time as the rest of the population (Lytle et al., 2015). This collective must adapt to the demands of their jobs, taking into account the age-related physical and cognitive changes they are undergoing (Mortensen et al., 2014; Kagan and Meléndez-Torres, 2015), which are often accompanied by changes in emotion regulation (Scheibe and Zacher, 2013) and motivational orientations (Kanfer and Ackerman, 2004). On another hand, organizations cannot allow themselves the luxury of losing these members, who accumulate a large amount of implicit knowledge and professional experience (Stoddart et al., 2014). Hence, both from the personal and organizational viewpoint, the study of adjustment to aging or of people's adaptation strategies to age-related physical and cognitive changes, and their relation to other psychosocial variables at work, deserves more detailed attention (Ekici and Koydemir, 2016).

## Adjustment to aging

The approach of Selection, Optimization and Compensation (SOC) has been proposed as a mechanism of successful adjustment to aging (Baltes and Baltes, 1990). It consists of diverse strategies that promote a positive balance between age-related losses and gains. Older people could implement three types of strategies: selecting the aspects of their lives that are important to them, optimizing the resources and tools that help them gain achievements in the selected aspects, and, lastly, compensating for the losses in those aspects, adapting to vital and environmental changes.

The recent meta-analysis on SOC strategies (Moghimi et al., 2017) briefly reported the evolution of the SOC strategies' assessment. Most organizational researchers relied on the original version of the Baltes et al. (1999) self- report questionnaire, and the short version (12 items) has been used most often. The instrument showed adequate psychometric properties (Zacher and Frese, 2011) and the meta-analysis conducted by Moghimi et al. (2017) summarized consistent relationships between SOC use and job performance measures (sample size-weighted *r* = 0.23 for self-reported, *r* = 0.21 for non- self-reported), based on 26 primary studies. Previous adaptation processes have been conducted in order to reduce the scale to nine items (Shang et al., 2015), or to adapt it to physically demanding work (Müller and Weigl, 2017). Empirical findings showed satisfying psychometric properties and adequate correlations between the original scale and the adapted versions. Furthermore, as SOC is focused on subjective experiences regarding aging, also adaptation processes to different cultures showed adequate results, as Japan, China or Sweden (Chou and Chi, 2001; Viglund et al., 2013; Okabayashi, 2014). The Japanese version showed adequate reliability coefficients, ranging from 0.67 to 0.82 for the different subscales (Okabayashi, 2014). Moreover, the Swedish version showed also adequate stability with a 2-week interval, and stable correlations with the self-esteem and coping questionnaires used in the study (Viglund et al., 2013). Finally, the Chinese version found satisfactory internal consistency coefficients ranging from 0.60 to 0.65 (Chou and Chi, 2001).

Hence, SOC strategies, as behaviors intended to cope with age-related changes in job resources, could later influence employees' outcomes. Despite the adaptation of the SOC questionnaire to different languages (Chou and Chi, 2001; Viglund et al., 2013; Okabayashi, 2014), there was no adapted version of this instrument in Spanish.

### Organizational citizenship behaviors and adaptive performance

Employee performance is of vital importance to an organization. To a large measure, the achievement of the company's goals depends on it. Regarding older workers, the decrease in their performance is one of the most debated aspects. Hence, it is interesting to appraise the relations between SOC and performance. On the one hand, modern organizations need their employees to be involved in the so-called organizational citizenship behaviors (hereafter, OCB). These consist of all the behaviors that are not specifically described as part of job performance but which are focused on the achievement of the organizational goals. Regarding older workers, the decrease in their performance at work is one of the most debated aspects. Hence, it is interesting to appraise the relations between SOC and organizational citizenship behaviors (hereafter, OCB). Moreover, the modern concept of performance has been expanded, introducing a novel dimension, adaptive performance (Koopmans et al., 2014), which is related to learning. As a recent meta-analysis showed (Moghimi et al., 2017) there is a positive relationships between SOC strategies and employee's desirable outcomes.

Despite the fact that age is a multidimensional concept (Kooij et al., 2008), chronological age has been the most widely used indicator in research given that in can be easily measured, is objective and affects everybody. Therefore, this study uses chronological age as an observable indicator of research purposes, following Aaltio et al.'s procedure 2014. Based on Martín (2005), we included participants above 40 years. This criterion seems to be in agreement with more recent findings. For example, employment rates among highly educated Europeans (ISCED 5 and 6) was more frequent for those aged under 39 years than for the group aged 40 and over (Eurydice, 2009). Moreover, according to the Eurobarometer Survey (2012), only 18% of Europeans believe that age discrimination against people aged 30 or younger is common, whereas 45% think that discrimination against people over 50 years old is widespread (TNS Opinion Social, 2012).

The current data set includes the assessment of the SOC-12 in a first sample of workers over 40, and in a second sample of workers over 45. It contributes to existing research by providing data from (a) two different samples of aged workers, (b) correlations with other theoretically related variables (adaptive performance and OCB), and (c) it provides socio-demographic data. Different criteria variables have been included in each data collection (adaptive performance and OCB) in order to offer the possibility to test the relationships between the SOC strategies and diverse measures of performance. It therefore enables researchers to further explore the associations between SOC-12 factors and aged workers attitudes and behaviors, as well as potential moderators of these associations (age, gender, etc.).

## Methods

### Ethical statement

The Ethical Committee of the third author's University Bio-ethical Committee of the National Distance Education University (UNED) approved the Project in May 2016.

### Participants

This study was carried out with two samples. The first one (Sample 1) included workers aged between 40 and 63 (*N* = 196), and the second one (Sample 2) was made up of workers aged between 45 and 57 years (*N* = 300). The mean age of Sample 1 58.8 years (*SD* = 2.38), 55% were males, and mean of job tenure in the company was 35.8 years (*SD* = 7.5). The mean age of Sample 2 was 53.3 (*SD* = 4.9), 45.7% were males, and the mean of job tenure in the company was 22.8 years (*SD* = 12.2). Regarding the type of job, in Sample 1, 3.7% were managers, 23.8% were technicians and middle managers, and 65% were employees. Regarding the professional sector, in Sample 1, 8% of the participants belonged to the banking sector, 6.4% to telecommunications, 9% to the production industry, and the rest to the services sector. In Sample 2, regarding the type of job, 6.7% were managers, 39.8% were technicians and middle managers, and 45% were employees. In Sample 2, regarding the professional sector, 3% of the participants belonged to the banking sector, 8.7% to telecommunications, 13% to the production industry, and the rest to the services sector.

### Procedure

We developed the Spanish version of the SOC-12 scale in two steps. First, the original 12-item SOC scale in English (Baltes et al., 1999) was translated to the Spanish context. The first and the last authors of the study, as experts in aging, drafted the items based on the original version in English. Next, back-translation was carried out by a native English speaker, who was unaware of the contents of the original scale. Then, the second and third author compared the outcome with the original version of the questionnaire. Discrepancies were resolved by reviewing the redaction since and adequate level of agreement has been reached. Secondly, we administered the SOC scale to the two samples that make up the present study (Sample 1: workers between 40 and 63, and Sample 2: workers between 45 and 57). The first data collection has been conducted during June–July 2016, while the second has been conducted during September-November 2016. This step of the study was carried out by means of questionnaires distributed in different organizations by the collaborators of the research team, who carried out the task after having received precise instructions to homogenize the administration procedures of the tests. The participants were informed of the goals of the study, of the anonymity of the data collected, and they expressed their consent, after which they completed the workbook containing the diverse scales of the study. All the questionnaires in both data collection procedures have been filled in a paper format, and the order of presentation of the scales has been counterbalanced.

### Instruments

#### SOC-12

The scale used is based on the SOC Questionnaire (Baltes et al., 1999), which includes 12 items in its original brief version (Please, see Table 1). It has four subscales: Elective Selection, Loss-Based Selection, Optimization, and Compensation. Participants were requested to rate the frequency of use of their specific strategies on a five-point Likert-type scale, which ranged from 1 (*Never*) to 5 (*Always*).

**Table 1:** Reliability analysis and exploratory factor analysis. Sample 1 (*N* = 196) and Sample 2 (*N* = 300).

**Items**	**Sample 1**										**Sample 2**			
	***M***	***SD***	***S***	***K***	***CIT***	***AIE***	***F 1***	***F 2***	***F 3***	***F 4***	***CIT***	***AIE***	**λ**	***t***
1. I keep working on what I have planned until I succeed. [Continúo trabajando en lo que me he propuesto hasta que lo consigo].	4.01	0.65	−0.34	0.44	0.39	0.78	0.82	0.10	0.04	0.05	0.52	0.77	0.86	29.45
2. I make every effort to achieve a given goal. [Hago todo el esfuerzo posible para alcanzar mis objetivos].	4.05	0.70	−0.51	0.45	0.46	0.77	0.76	0.06	0.23	0.11	0.53	0.77	0.88	14.51
3. If something matters to me, I devote myself fully and completely to it. [Si algo es importante para mí, me dedico a ello totalmente].	3.86	0.72	−0.37	0.55	0.46	0.77	0.63	0.18	0.34	0.06	0.45	0.78	0.73	7.46
4. When things don't go as well as before, I choose one or two important goals. [Si no puedo seguir adelante como solía hacerlo, dirijo mi atención primero al objetivo más importante].	3.53	0.89	−0.62	0.19	0.48	0.77	0.10	0.86	0.03	0.19	0.48	0.78	0.75	3.26
5. When I can't do something important the way I did before, I look for a new goal. [Cuando algo es totalmente imposible de conseguir, dirijo mi esfuerzo a lo que aún posible].	3.73	0.80	−0.82	1.29	0.53	0.77	0.29	0.77	0.09	0.21	0.40	0.79	0.94	2.94
6. When I can't do something as well as I used to, I think about what exactly is important to me. [Si no puedo hacer algo tan bien como solía hacerlo, me concentro sólo en lo esencial].	3.09	0.89	−0.14	−0.38	0.39	0.78	0.16	0.76	0.36	0.09	0.39	0.79	0.53	2.03
7. I always focus on the one most important goal at a given time. [Siempre me concentro en un objetivo importante a la vez].	3.41	0.76	0.14	0.08	0.47	0.77	0.12	0.14	0.78	0.07	0.43	0.78	0.87	9.15
8. When I think about what I want in life, I commit myself to one or two important goals. [Me comprometo con uno o dos objetivos importantes en mi vida].	3.38	0.92	−0.15	−0.40	0.52	0.77	0.11	0.12	0.75	0.25	0.47	0.78	0.68	2.86
9. I concentrate all my energy on few things [Siempre persigo mis metas una a una].	3.36	0.90	−0.27	0.02	0.44	0.78	0.25	0.03	0.69	0.03	0.39	0.79	0.76	4.75
10. When something doesn't work as well as usual, I keep trying other ways until I can achieve the same result I used to. [Cuando algo no me sale como siempre, continúo intentando otros caminos para alcanzar el resultado al que estoy acostumbrado].	3.45	0.80	−0.26	0.11	0.39	0.78	0.07	0.14	0.05	0.82	0.42	0.78	0.79	4.08
11. When something in my life isn't working as well as it used to, I ask others for advice or help. [Cuando algo en mi vida no me sale como siempre, pido ayuda o consejo a otros].	3.39	0.87	−0.03	−0.26	0.48	0.77	0.08	0.20	0.25	0.80	0.41	0.79	0.72	3.08
12. When it becomes harder for me to get the same results, I keep trying harder until I can do it as well as before. [Cuando se me hace difícil lograr los mismos resultados, continúo intentándolo hasta que puedo hacerlo tan bien como antes].	4.10	0.66	−0.22	−0.32	0.39	0.79	0.29	0.09	0.09	0.63	0.44	0.78	0.81	5.92

*M, Mean; SD, Standard deviation; S, Skewness; K, kurtosis; CIT, Correlation item-total; AIE, Alpha if item is deleted*.

*F1, Optimization; F2, Loss-based Selection; F3, Elective Selection; F4, Compensation*.

#### Adaptive performance

We used the *Adaptive Performance* subscale of the Spanish version (Pérez-Larrazabal, 2016) of the Individual Work Performance questionnaire (IWP; Koopmans et al., 2014). It includes six items that assess the employee's capacity to adapt to the changes demanded by the occupational role or the work environment. Example items are: “*I have coped with stress, difficult situations and adversities*,” “*I have come up with creative solutions to novel, difficult problems*,” “*I have adjusted work goals when necessary*”. Reliability was α = 0.74, and it was α = 0.86 in the present study.

#### Organizational citizenship behaviors

We used the Spanish Organizational Citizenship Behavior scale (Dávila de León and Finkelstein, 2016), providing a global measure of the behaviors aimed at benefiting peers and companies, with 16 items. Example items are: “*I show interest in the image of the organization”* and “*I dedicate time to help others who have problems related or not to the tasks.”* The Likert-type response scale ranged from 0 (*Not at all*) to 4 (*Always or almost always*). The reliability analysis showed satisfactory internal consistency for the scale in a previous study (α = 0.79) (Dávila de León and Finkelstein, 2016).

### Data analyses

Descriptive analysis of the items and exploratory factor analysis (EFA) were conducted with Sample 1, using SPSS 24, and confirmatory factor analysis (CFA) was performed with Sample 2, using Amos 24. Cronbach's alpha and the CFA indices were used to appraise the internal consistency of the SOC-12 scale in both samples. Lastly, relationships between the SOC-12 and the IWP and OCB scales have been tested.

### Exploratory and confirmatory factor analysis

Firstly, all the items showed levels of skewness lower than 1 and levels of kurtosis lower than 2. In addition, item-total correlations varied between 0.63 and 0.39. For the EFA, the Kaiser-Meyer-Olkin index (KMO = 0.75) and Bartlett's sphericity test (χ^2^ = 583.3, *p* < 0.000) indicated the suitability of the data. The EFA was performed with the principal components procedure and varimax rotation. The Kaiser criterion supported the 4-fimensional solution, with eigenvalues >1. The four factors explained 65.9% of the total variance. It was found that each item had a loading higher than.41 on its factor and loadings lower than 0.39 on any of the other factors.

We performed a CFA with Sample 2 in order to replicate the factor solution obtained by EFA, using the maximum likelihood procedure χ^2^ = 129.85; *p* = 0.00; d.f. = 48; χ^2^/d.f = 2.7; GFI = 0.94; AGFI = 0.90; CFI = 0.92; IFI = 0.92; RMR = 0.04; RMSEA = 0.07). The consideration of alternative hypotheses is considered an important stage that contributes evidence to support the internal structure of a scale (Morata et al., 2015). For this purpose, the fit of the four-factor model was compared with three successive models, of three (χ^2^ = 309.17; *p* = 0.00; d.f. = 51; χ^2^/d.f = 6.1; GFI = 0.85; AGFI = 0.77; CFI = 0.73; IFI = 0.74; RMR = 0.07; RMSEA = 0.13, Δχ^2^ = 179.3), two χ^2^ = 384.88; *p* = 0.00; d.f. = 0.53; χ^2^/d.f = 7.3; GFI = 0.81; AGFI = 0.72; CFI = 0.66; IFI = 0.66; RMR = 0.07; RMSEA = 0.14, Δχ^2^ = 75.71), and a single factor χ^2^ = 464.93; *p* = 0.00; d.f. = 54; χ^2^/d.f = 8.6; GFI = 0.78; AGFI = 0.68; CFI = 0.57; IFI = 0.58; RMR = 0.08; RMSEA = 0.163, Δχ^2^ = 80.05) for the same data. As the number of factors was reduced, the fit indexes progressively worsened. Therefore, we rejected the more parsimonious model and chose the four-factor solution.

### Reliability

Cronbach alpha coefficients of the global SOC-12 scale (α = 0.80) and of the four subscales were appropriate. These values were confirmed in Sample 2 (global SOC-12 α = 0.80). To appraise individual reliability of each item we used their factor loadings in the CFA or lambdas (λ), which can be seen in Table 1. The reliability of the global scale is also evaluated through the composite reliability (CR). Factor 1 (*Optimization*) achieved a CR of 0.87, the CR of Factor 2 (*Loss-based selection*) reached 0.80, that of Factor 3 (*Elective selection*) was 0.82, and the CR of Factor 4 (*Compensation*) was 0.82. The correlation between the subscales in Sample 1 was moderate, while in Sample 2, the highest correlation between subscales was even lower. Lastly, it was found that the four factors of the SOC-12 were significantly associated with adaptive performance and organizational citizenship behaviors both in Sample 1 and in Sample 2, as Table 2 shown.

**Table 2:** Correlation matrices among SOC-12 subscales, adaptive performance and organizational citizenship behaviors.

**Variables**	**1**	**2**	**3**	**4**	**5**	**6**
1. Factor 1 *Optimization*	–	0.28^**^	0.46^**^	0.33^**^	0.45^**^	0.46^**^
2. Factor 2 *Selection by loss*	0.28^**^	–	0.37^**^	0.32^**^	0.12	0.08
3. Factor 3 *Elective Selection*	0.39^**^	0.26^**^	–	0.38^**^	0.28^**^	0.24^**^
4. Factor 4 *Compensation*	0.36^**^	0.27^**^	0.31^**^	–	0.27^**^	0.28^**^
5. Adaptive performance	0.43^**^	0.11	0.28^**^	0.28^**^	–	0.64^**^
6. Organizational citizenship behaviors	0.16^**^	−0.02	0.20^**^	0.22^**^	0.22^**^	–

*Sample 1 above diagonal, Sample 2 below diagonal*.

** *p < 0.01*.

Next, using linear and multiple hierarchical regression analysis, we found that the SOC-12 factors predicted the participants' adaptive performance and organizational citizenship behaviors. The factors were introduced in a single step that reached statistical significance. The results show that the different factors have different predictive power as a function of the type of results included in the regression. Regarding organizational citizenship behaviors, the results are disparate, as the percentage of explained variance only reached 8% in Sample 2, that is, in the workers between 45 and 57 [Adj. *R*^2^ = 0.07, *F*_(4, 295)_ = 6.53, *p* < 0.001) but it increased to 24% in Sample 1, workers aged between 40 and 63 [Adj. *R*^2^ = 0.23, *F*_(4, 191)_ = 15.15, *p* < 0.001]. Lastly, the percentage of explained variance in adaptive performance reached 23% in Sample 1 [Adj. *R*^2^ = 0.21, *F*_(4, 191)_ = 13.68, *p* < 0.001] and 22% in Sample 2 [Adj. *R*^2^ = 0.21, *F*_(4, 295)_ = 20.34, *p* < 0.001].

### Suggestion of future avenues of research using this data set

Only a limited number of studies have utilized quantitative instruments to evaluate SOC at work; and no studies have applied the SOC to Spanish population. Compared with the original English version, the Spanish version of the SOC-12 showed adequate psychometric properties. Our data suggest that the strategies used by workers are an adequate tool to address their age-related reduction of energy and attentional resources that could lead to reducing desirable outcomes, such as OCB and adaptive performance. Concerning the size and representativeness of the samples, the limitations of this data are obvious, especially those due to the sampling procedure used. Moreover, all the data proceed from self-reports, which can include a source of uncontrolled error from the common variance. However, because SOC is focused on subjective expectations regarding aging, deviations from external criteria would not necessarily indicate that the SOC is an invalid instrument. Instead, these differences suggest sources of bias in aged workers' expectations, and ways for counseling and intervention. Also, some aspects should be taken into account when readers reuse the data. For instance, previous levels of psychological wellbeing and performance could affect aged workers adaptation to aging (Linares et al., 2017). Although the findings of this work are limited, organizations, HR managers and workers themselves may adopt procedures to positively influence employee's adaptive performance and OCB (Müller and Weigl, 2017). In the same sense, the influence of the organizational environment should be included as a control variable (Zacher and Yang, 2016). Related to outcome variables, more objective measures could be included, such as health, assessed through number of diseases or medical interventions, as suggested (Zacher and Schmitt, 2016). In summary, we conclude that the available data can be used with sufficient guarantee in order to expand research on predictors of successful aging and empirically support further theoretical development. In this regard, future analyses could reuse the data in order to develop practical implications for people who possess fewer resources because they could be in a worse position to prepare them for coping with future loss (Annink et al., 2016). Firstly, due to the fact that the literature documents the existence of gender-specific patterns of stress and coping strategies (Lee and Mason, 2014), the exploration about gender invariance of successful aging at work measures is a topic of interest (Kocalevent et al., 2014). Secondly, future findings support more recent proposals such as job-crafting, as employees who are proactively involved in increasing their arsenal of resources for the task will be able to cope more efficiently with unhealthy working environments, and to engage in productive roles (Vignoli et al., 2017). As older workers are often members of stigmatized groups and they are frequently prevented from using their current resources, organizational interventions based in the data analysis should be aim at overcoming the negative stereotyping at work would protect them from being less able to use their existing resources (Lytle et al., 2015).

### Dataset description

The data set, called successful aging at work, is deposited in Figshare repository and is accessible through the following hyperlink: https://figshare.com/articles/Adjustment_to_aging/5597884

The file named Sample 1 (.sav) contains individual responses for the 196 participants on the four factors of the SOC-12 scale, adaptive performance assessment along with demographics data (age and gender). The file named Sample 2 contains the 300 individual responses (.sav) for the four factors of the SOC-12 scale and Organizational Citizenship behaviors, along with the demographics data.

## Author contributions

All the authors contributed equal to the manuscript, collecting the data, conducting the analyses, and writing the manuscript.

### Conflict of interest statement

The authors declare that the research was conducted in the absence of any commercial or financial relationships that could be construed as a potential conflict of interest.
